# Isothermal Crystallization Kinetics of Poly(ethylene oxide)/Poly(ethylene glycol)-*g*-silica Nanocomposites

**DOI:** 10.3390/polym13040648

**Published:** 2021-02-22

**Authors:** Xiangning Wen, Yunlan Su, Shaofan Li, Weilong Ju, Dujin Wang

**Affiliations:** 1 Key Laboratory of Engineering Plastics, CAS Research/Education Center for Excellence in Molecular Sciences, Institute of Chemistry, Chinese Academy of Sciences, Beijing 100190, China; xnwen@iccas.ac.cn (X.W.); sfli507@iccas.ac.cn (S.L.); wlju507@iccas.ac.cn (W.J.); djwang@iccas.ac.cn (D.W.); 2 University of Chinese Academy of Sciences, Beijing 100049, China

**Keywords:** poly(ethylene oxide), nanocomposites, grafted silica nanoparticles, isothermal crystallization kinetics

## Abstract

In this work, the crystallization kinetics of poly(ethylene oxide) (PEO) matrix included with poly(ethylene glycol) (PEG) grafted silica (PEG-*g*-SiO_2_) nanoparticles and bare SiO_2_ were systematically investigated by differential scanning calorimetry (DSC) and polarized light optical microscopy (PLOM) method. PEG-*g*-SiO_2_ can significantly increase the crystallinity and crystallization temperature of PEO matrix under the non-isothermal crystallization process. Pronounced effects of PEG-*g*-SiO_2_ on the crystalline morphology and crystallization rate of PEO were further characterized by employing spherulitic morphological observation and isothermal crystallization kinetics analysis. In contrast to the bare SiO_2_, PEG-*g*-SiO_2_ can be well dispersed in PEO matrix at low *P*/*N* (*P*: Molecular weight of matrix chains, *N*: Molecular weight of grafted chains), which is a key factor to enhance the primary nucleation rate. In particular, we found that the addition of PEG-*g*-SiO_2_ slows the spherulitic growth fronts compared to the neat PEO. It is speculated that the interfacial structure of the grafted PEG plays a key role in the formation of nuclei sites, thus ultimately determines the crystallization behavior of PEO PNCs and enhances the overall crystallization rate of the PEO nanocomposites.

## 1. Introduction

Polymer nanocomposites (PNCs) [1,2,3,4,5] have gained considerable interest over the past decades. The material properties of semi-crystalline polymers are closely related to the morphology of nanoparticles (NPs) [6,7,8,9]. A convenient method to improve the dispersion of NPs in the polymer matrix is to graft polymer chains on the NPs surface [10,11]. The addition of polymer grafted nanoparticles (PGNPs) into polymer matrix can optimize their mechanical properties [12,13], optical ability [14,15], rheological properties [16,17], and electrical performance [18,19]. Recent studies have shown the spatial distribution of PGNPs can be controlled by varying the grafting density (*σ*), chain length of matrix vs. the grafted polymer (*P*/*N*), NPs size, and chemical properties of the grafted chains [20,21]. 

In semi-crystalline nanocomposites, the presence of NPs can significantly impact the crystallinity, crystal nucleation, and growth of polymer matrix [22,23,24], which is vital to fully exploit the potentially excellent properties of PNCs. Over the past decades, there have been extensive works on the crystallization kinetics of polymer nanocomposites containing various one-dimensional and two-dimensional PGNPs, i.e., carbon nanotubes, cellulose nanocrystal, clay and graphene oxide [25,26,27,28] as well as how they were modulated by the PGNPs addition. Müller et al. [27] found that the multiwall carbon nanotubes grafted linear poly(ε-caprolactones), (PCL) (MWNT-*g*-PCL) can nucleate the linear PCL but cause a decrease both in spherulitic growth rate and in the overall isothermal crystallization kinetics of cyclic PCL. The results line in the contact between liner grafted chains with cyclic PCL, forming a transient entanglement network, thus slowing the crystallization rate. It seems the interfacial interactions and chain dynamics are the main factors affecting the crystallization of PNCs. 

Recently, increasing attention has focused on the PNCs with three-dimensional PGNPs, i.e., silica (SiO_2_), in which the grafted chains show more controllability and richer conformational behavior than the case of flat surface [29]. Wen et al. [30] demonstrated that controlling the spatial dispersion of PEG-*g*-SiO_2_ in PEO matrix can presumably modulate the crystallization behavior of the matrix chains. PEG-g-SiO_2_ (in the case of high σ and low *P*/*N*) can significantly increase the nucleation efficiency of PEO, where the grafted NPs are under a good dispersion state. The aggregation of PEG-g-SiO_2_ at higher *P*/*N* values and low σ occurs to limit the effectiveness of grafted chains on the nucleation ability of the nanocomposites. Jimenez et al. [13] study the crystallization kinetics of PEO matrix with amorphous poly(methyl methacrylate) chains grafted SiO_2_ (PMMA-*g*-NPs). It was found the crystal nucleation is unaffected by the addition of PMMA-*g*-NPs, while causing a decrease in spherulitic growth, crystallinity, and melting points. NPs functionalized with either unimodal or bimodal amorphous polymer chains exhibit various self-assembly morphologies [31] and also show retardation in spherulitic growth rates. The current understanding considers these reductions mainly come from two aspects: (i) The increase in viscosity with the inclusion of PGNPs [31]. (ii) The confinement effects imposed on the polymer melts due to the addition of NPs [13]. Both of these effects slow down the chain mobility to crystal growth front. 

PEO is an attractive semi-crystalline polymer in many fields of research [32], and its silica nanocomposites can enhance the ion conductivity for applications in biomaterials and as electrolytes in lithium batteries [33]. In our previous study [34], the semi-crystalline PEG chains grafted SiO_2_ (PEG-*g*-SiO_2_) endows a notable increase in the overall crystallization rate. The results indicate that the interfacial structure is closely related to the grafting density, which plays a critical role in the nuclei formation and finally determines the non-isothermal crystallization kinetics of the PNCs. The addition of grafted silica NPs can also enhance nucleation of semi-crystalline polymer matrix like PCL [35], poly(L-lactide) (PLLA) [36], polypropylene (PP) [37], etc. 

In order to understand the parameters affecting the role of semi-crystalline chains grafted NPs on the isothermal crystallization kinetics of polymer, the influence of PEG-*g*-SiO_2_ on the nucleation kinetics, morphology, spherulitic growth rate, and overall crystallization kinetics of PEO nanocomposites was systematically investigated in this work. Here, PEG-*g*-SiO_2_ with a grafting density of 0.73 chains/nm^2^ was added into two different PEO matrices (i.e., the molecular weights of matrix PEO is 1700 and 7800 g⋅mol^−1^, respectively). We found that the presence of PEG-*g*-SiO_2_ remarkably elevates the nucleation density and crystallization rate, especially in the case of a better dispersion (at low *P*/*N*). These results are different from the earlier studies where the grafted chains are amorphous [13,31]. This work, therefore, aims to elucidate the crystallization kinetics in the polymer nanocomposites with semi-crystalline chains grafted NPs.

## 2. Experimental

### 2.1. Materials

Methoxy polyethylene glycol (MPEG) with molecular weights *M_n_* = 4 kg⋅mol^−1^, was purchased from TCI (Tokyo, Japan). Monodisperse spherical SiO_2_ nanoparticles with a mean diameter = 50 nm, were prepared by the method of Stöber and Fink [38]. N-(2-aminoethy)-3-aminopropylmethyldimethoxysilane (ADMS) was purchased from Alfa Aesar Co., Shanghai, China. Poly (ethylene oxide) with molecular weights of 1700 and 7800 g⋅mol^−1^ were purchased from Polymer Source, Inc (Montreal, QC, Canada).

### 2.2. Sample Preparation

Monodisperse SiO_2_ were separately grafted with PEG chains of *M_n_* = 4 kg⋅mol^−1^ through a series of experiments. The grafting densities (*σ* values in chains/nm^2^ were calculated by TGA) obtained using the “grafting to” method, which has been reported in our previous study [30,39]. For clarity, the PEG grafted SiO_2_ was denoted as PEG-*g*-SiO_2_, where the *σ* in this study is 0.73 chains/nm^2^.

To prepare the PEO/PEG-*g*-SiO_2_ nanocomposites, PEO and PEG-*g*-SiO_2_ were individually dispersed in acetonitrile at room temperature and then mixed in the desired volume ratios to obtain the PNCs with a SiO_2_ content of 24 wt%. The mixtures were sonicated for 5 min and then stirred for ≈ 6 h at room temperature before casting onto Petri dishes. The nanocomposites were dried under a fume hood for 24 h to remove the solvent.

### 2.3. Characterization

#### 2.3.1. Thermogravimetric Analysis

The *σ* of PEO was calculated by a PerkinElmer 8000 thermogravimetric analysis apparatus [40] (TGA, PE8000, Waltham, MA, USA). Samples of 2–3 mg were heated from 50 to 100 °C at a rate of 40 °C/min and held for 2 min at 100 °C to remove physically adsorbed water, then heated from 100 to 800 °C with a rate of 20 °C/min.

#### 2.3.2. Differential Scanning Calorimetry

The non-isothermal crystallization and melting behavior of PEO nanocomposites were recorded by a PerkinElmer 8500 DSC apparatus (Waltham, MA, USA). The equipment was calibrated with indium and tin standards. The samples (3–5 mg) were encapsulated in aluminium pans, and ultra-pure nitrogen was used as a purge gas. First, the samples were heated to 80 °C and held for 3 min at that temperature to erase any previous thermal history. Second, they were cooled to −60 °C, and finally, reheated to 80 °C. All tests were performed at a cooling and heating rate of 10 °C/min. The peak temperatures of the obtained crystallization (*T_c_*) and melting (*T_m_*) exotherms were recorded.

The isothermal crystallization of PEO nanocomposites was recorded by PerkinElmer 8500 DSC under a N_2_ atmosphere. The samples were held for 3 min at 80 °C to erase thermal history, then cooled at 100 °C/min to the selected crystallization temperature and held for 20 min.

The glass temperature (*T_g_*) of the PEO nanocomposites was recorded by PerkinElmer 8500 DSC under a He atmosphere. The samples were cooled to −150 °C after holding 3 min at 80 °C under a ballistic cooling procedure, approximately with a rate of 280 °C/min. Then heated to 0 °C at 500 °C/min, and the *T_g_* was recorded during subsequent heating scans.

#### 2.3.3. Polarized Light Optical Microscope

A polarized light optical microscope (PLOM, Olympus BX51, Tokyo, Japan) equipped with a Linkam THMS600 temperature controller was used to observe the crystalline morphology of PNCs. The samples were sandwiched between two cover glasses and heated to 80 °C for 5 min. Then, the samples were cooled at 60 °C/min to desired *T_c_*, and the number of spherulites and their sizes were monitored as a function of time. The nucleation density (N*) was calculated from the numbers by determining the volume (cm^3^) from the measured sample thickness and the area of the field of view of the microscope.

## 3. Results and Discussion

### 3.1. Nucleation Kinetics of PEO Nanocomposites Studied by PLOM: Primary Nucleation

**Figure 1:**
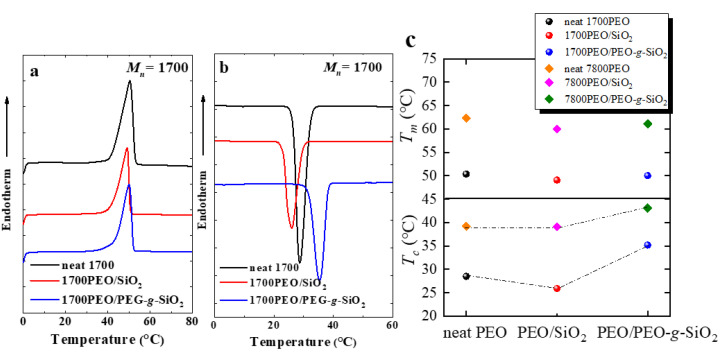
DSC heating (**a**,**b**) cooling scans of neat PEO, PEO/SiO_2_, and PEO/PEG-*g*-SiO_2_ with a matrix molecular weight of 1700 g/mol. (**c**) The changes in the *T_c_* and *T_m_* for the two different nanocomposites with matrix molecular weights of 1700 g/mol and 7800 g/mol, and the silica content is 24 wt% in the nanocomposites studied here.

**Figure A1:**
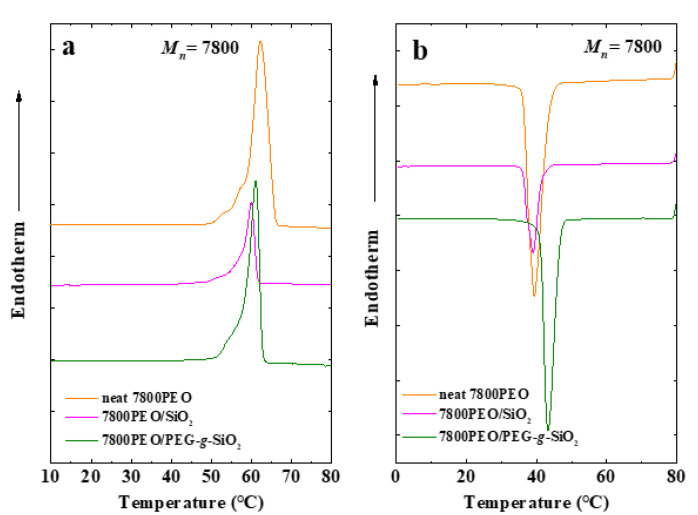
DSC heating (**a**,**b**) cooling scans of neat PEO, PEO/SiO_2_, and PEO/PEG-g-SiO_2_ with a matrix molecular weight of 7800 g/mol.

Before exploring the isothermal crystallization kinetics in PEO nanocomposites, we first focus on the non-isothermal crystallization behavior of the samples. Figure 1a,b illustrates the DSC melting and cooling curves of neat PEO, PEO/SiO_2_ and PEO/PEG-*g*-SiO_2_ with a matrix molecular weight (*M_n_*) of 1700 g/mol and the SiO_2_ content of 24 wt% (The DSC curves of PEO nanocomposites with matrix *M_n_ =* 7800 g/mol are shown in Figure A1 of Appendix A). Figure 1c presents the crystallization (*T_c_*) and melting (*T_m_*) temperatures of all PEO nanocomposites employed here. The appearance of the increased *T_c_* in PEO/PEG-*g*-SiO_2_ suggests that the PGNPs exhibit a significant nucleation effect on the crystallization process of PEO. Based on our recent work [30], it is probably more relevant to the better dispersion state (the dispersion state of PEO nanocomposites was studied using SAXS scattering combined with TEM, not shown here) of the PGNPs under a higher grafting density (*σ* = 0.73 chains/nm^2^) and lower *P*/*N* value (the *P*/*N* studied here is 0.425 and 1.95) compared to the bare SiO_2_. The well dispersed sample, 1700PEO/PEG-*g*-SiO_2_ (where 1700 represents the matrix *M_n_* = 1700 g/mol), showed a more excellent increase in *T_c_* (neat 1700PEO undergoes crystallization at 28.5 °C and 1700PEO/PEG-*g*-SiO_2_ at 35.2 °C) as well as an increase in the PEO crystallinity (Table 1 summarizes the relative parameters obtained in the crystallization process). The data in Figure 1c show there are minor differences in the melting point with the changing of *P*/*N*.

**Figure 2:**
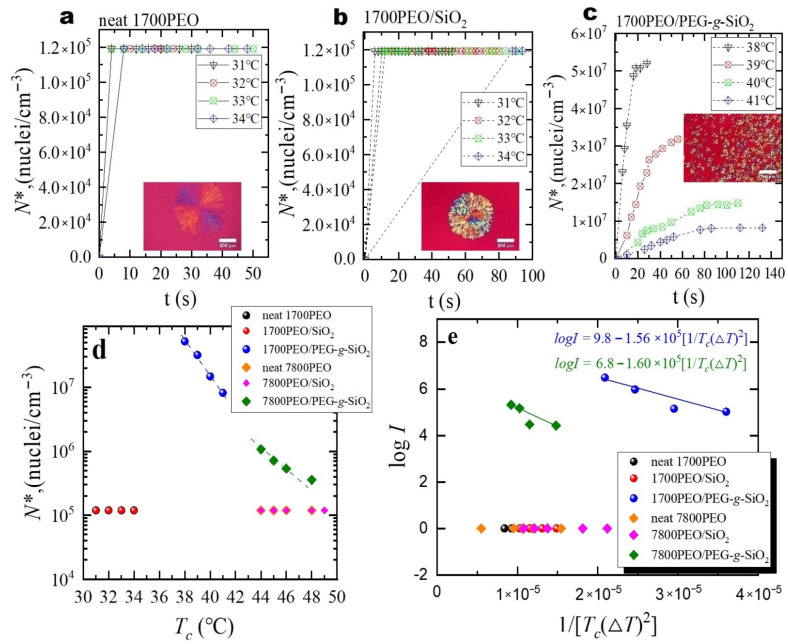
Nucleation density as a function of time at each crystallization temperature (**a**) neat PEO (**b**) PEO/SiO_2_, (**c**) PEO/PEG-*g*-SiO_2_. The inset images represent the PLOM images of PEO nanocomposites corresponding to 34 °C for neat PEO, 32 °C for PEO/SiO_2_ and 39 °C for PEO/PEG-*g*-SiO_2_. The matrix molecular weight of PEO is 1700 g/mol. (**d**) Nucleation densities of neat PEO, PEO/SiO_2_, and PEO/PEG-*g*-SiO_2_ at different *T_c_*. (**e**) Plots of log*I* versus 1/[*T_c_*(Δ*T*)^2^], the solid line is the fitting according to Equation (1).

**Figure A2:**
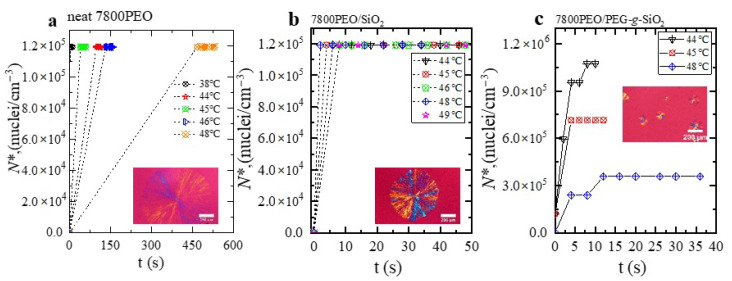
Nucleation density as a function of time at each crystallization temperature (**a**) neat PEO, (**b**) PEO/SiO_2_, (**c**) PEO/PEG-g-SiO_2_. The insets represent the PLOM images of PEO nanocomposites and the molecular weight of matrix PEO is 7800 g/mol.

In this part of the study, the main point is to defer a detailed exploration of the crystal nucleation and growth kinetics in PEO nanocomposites. Therefore, we employed PLOM measurements to monitor the spherulitic growth for different nanocomposites at different crystallization temperatures, *T_c_*. Figure 2 summarizes the primary nucleation kinetics studied by PLOM. The direct information on the nucleation ability of PEO nanocomposites can be obtained by counting the number of spherulites with time changing, as shown in Figure 2a–c. PEO and PEO/SiO_2_ exhibit similar nucleation kinetics with respect to the measured nucleation density, N* (as shown in Figure 2d). In the case of PEO/PEG-*g*-SiO_2_, PEG-*g*-SiO_2_ behaves more effectively as the nucleating agent and presents a much higher nucleation density in the *T_c_* range, i.e., 38–41 °C for 1700PEO/PEG-*g*-SiO_2_ (with *P*/*N* = 0.425) and 44–48 °C for 7800PEO/PEG-*g*-SiO_2_ (with *P*/*N* = 1.95) (details on the N* changing at matrix *M_n_* = 7800 g/mol are shown in Figure A2, Appendix B). It can be seen the nucleation densities of 1700PEO/PEG-*g*-SiO_2_ are almost two orders of magnitude higher than that of neat PEO, which shows a constant nucleation density in the *T_c_* range (as shown in Figure 2d). A similar changing tendency with higher nucleation densities can also be observed in 7800PEO/PEG-*g*-SiO_2_. This leads us to speculate that the grafted PEG chains may serve as a template providing an increase of the nucleation sites to enhance the N* of PEO nanocomposites. Regardless, the changing tendency of increasing nucleation density with a decrease of *P*/*N* is consistent with our recent study [30], the more stretched grafted PEG chains at high grafting density (0.73 chains/nm^2^) and lower *P*/*N* can enhance the interaction with matrix PEO, thus improving the nucleation density.

**Figure A3:**
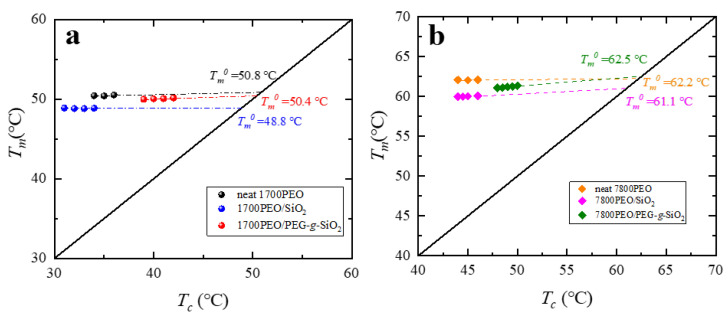
The equilibrium melting temperature of the PEO PNCs: (**a**) matrix M_n_ = 1700 g/mol, (**b**) matrix M_n_ = 7800 g/mol.

The primary nucleation rate *I* was obtained by counting the number of spherulites in a specific area at different crystallization times [41] (i.e., $I=\frac{dN^{*}}{dt}$). Turnbull–Fisher model [42,43] is adopted here to better understand the effects of NPs on the primary nucleation:

(1) $$\log I=\log I_{0}-\frac{\Delta F^{*}}{2.3kT}-\frac{16\sigma \sigma_{e}\left(\Delta \sigma \right)T_{m}^{0^{2}}}{2.3kT{\left(\Delta T\right)}^{2}{\left(\Delta H_{v}\right)}^{2}}$$

where *I_0_* is related to the segments’ diffusion from the melt state to the nucleation site. ΔF* represents a parameter proportional to the free energy of primary nucleation. *k* is 1.381 × 10^−23^ J·K^−^^1^. Δ*H_v_* is the volumetric melting enthalpy (J/cm^3^), and Δ*H_v_* can be calculated as Δ*H_v_* = Δ*H_m_*^0^·ρ (Δ*H_m_*^0^ is the melting enthalpy of 100% crystalline PEO [44] with a value of 205 J·g^−1^ and ρ is the monomer density of PEO with a value of 1.064 g·cm^−3^). Δ*T* is the supercooling calculated by Δ*T* = *T_m_*^0^ − *T_c_*, and *T_m_*^0^ is the equilibrium melting point. The *T_m_*^0^ of PEO nanocomposites studied here were determined by DSC 8500, as shown in Figure A3 of Appendix C. In PEO nanocomposites with matrix *M_n_* = 1700 g/mol and 7800 g/mol, it is observed that either isothermal thickening to the integral-folding chain (IF) (*n* = 0) crystal or thinning to the IF (*n* = l) crystal occurs depending upon the thermodynamic stability of the nonintegral-folding chain (NIF) crystal. Both thickening and thinning processes are observed at intermediate crystallization temperatures. An almost constant melting temperature may basically be attributed to the competition between overall crystallization and the isothermal thinning process (Appendix C) [45]. σ and σ_e_ are the free energies of the lateral and fold surface of PEO, respectively. Δσ is a parameter related to nucleation efficiency [43]. σσ_e_(Δσ) = 140 erg^3^/cm^6^ for 1700PEO/PEG-g-SiO_2_ and σσ_e_(Δσ) = 134 erg^3^/cm^6^ for 7800PEO/PEG-*g*-SiO_2_ were obtained from the slope of the straight line given by *logI* vs. 1/(*T*Δ*T*)^2^ [43].

Two basic conclusions can be obtained from Figure 2e: (1) The significant change of primary nucleation rate *I* is closely related to the addition of PGNPs, (2) nucleation rate decreases with the increase of *P*/*N* value in the measured temperature range. This result implies that the good compatibility between PEG-*g*-SiO_2_ and the PEO matrix at low *P*/*N* can enhance the interactions between matrix chains and PGNPs. Compared to the bare SiO_2_, the improvement of dispersion state of PEG-*g*-SiO_2_ appears to be the key factor for the enhancement of N* observed in Figure 2. Similar trends of increasing nucleation density have been observed in PLLA nanocomposites containing PEG grafted graphene oxide [46] and linear PCL system containing MWNT-*g*-PCL (linear PCL chains grafted multiwall carbon nanotubes) [27].

### 3.2. Growth Kinetics of PEO Nanocomposites Studied by PLOM: Secondary Nucleation

**Figure 3:**
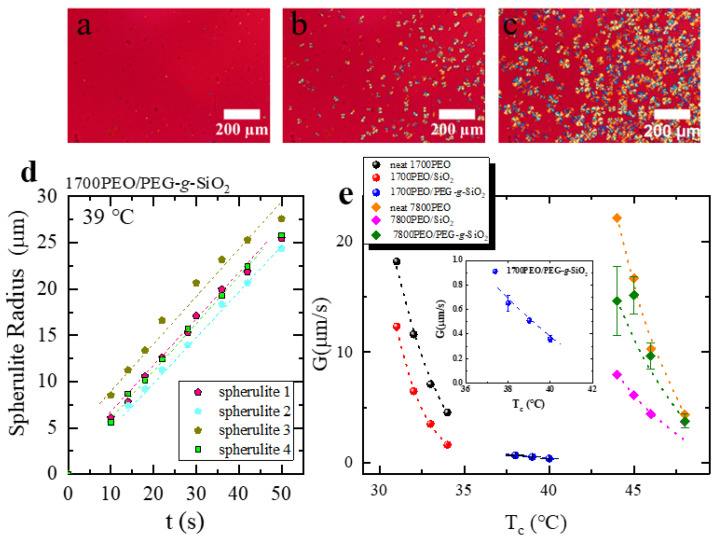
(**a**–**c**) PLOM images of 1700PEO/PEG-g-SiO_2_ isothermally crystallized at 39 °C taken at 18 s, 30 s, and 74 s, respectively. (**d**) The variations of spherulitic radius with a function of time for four selected spherulites. (**e**) Spherulitic growth rate, G, as a function of temperature for each of the indicated PEO nanocomposites. The dotted lines correspond to the Lauritzen–Hoffman fits. The insert is the enlarged image of 1700PEO/PEG-g-SiO_2_.

**Figure A4:**
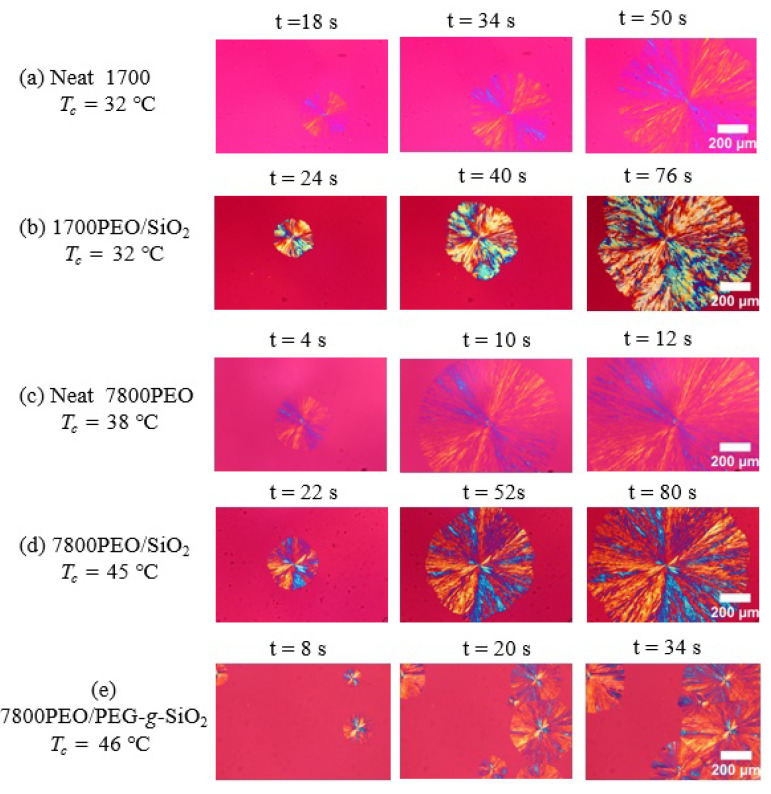
Spherulitic growth studied by PLOM isothermally crystallized at different *T_c_*. (**a**) neat1700 PEO, (**b**) 1700PEO/SiO_2_, (**c**) neat 7800PEO, (**d**) 7800PEO/SiO_2_, (**e**) 7800PEO/PEG-g-SiO_2_.

Following the discussions above, to further separate out the effects of PEG-*g*-SiO_2_ on the spherulitic growth kinetics (i.e., secondary nucleation), the growth process of each sample at different times is measured by PLOM. Figure 3a–c shows the micrographs of 1700PEO/PEG-g-SiO_2_ spherulites isothermally crystallized at 39 °C at different times. The results clearly show that the number of spherulites in 1700PEO/PEG-*g*-SiO_2_ increases with the increase of time, while neat PEO and PEO/SiO_2_ exhibit only one nucleus during the growth process, as shown in Figure A4 of Appendix D.

The linear dependence of the spherulitic radius on the measured time was shown in Figure 3d, which indicates there is no disturbance by the diffusion during growth.

**Figure A5:**
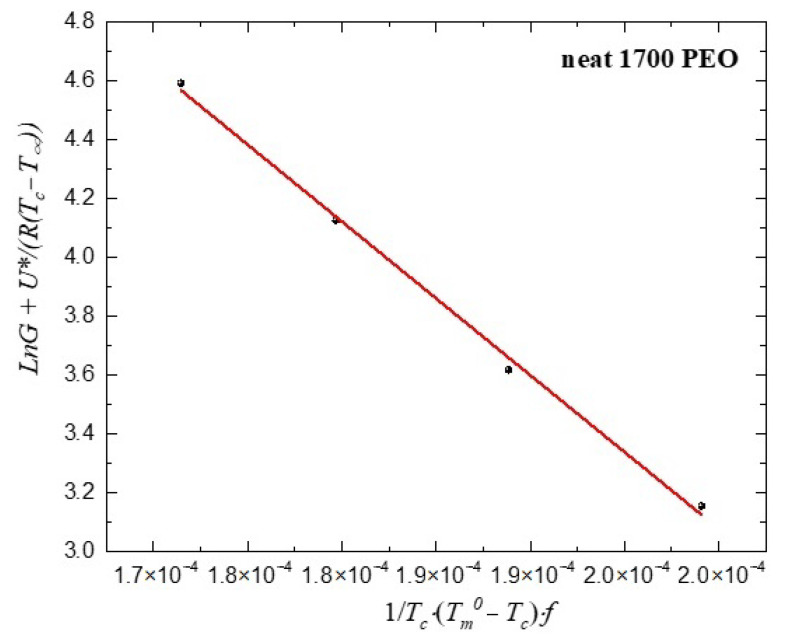
Spherulitic growth rate of neat PEO with M_n_ = 1700 g/mol measured by PLOM and the data were fitted by Lauritzen−Hoffman model (the solid line).

Lauritzen and Hoffman model (LH theory) is used here to ascertain the spherulitic growth rate (*G*) according to the following form [47,48,49]:

(2) $$G\left(T\right)=G_{0}\exp(\frac{-U*}{R\left(T_{c}-T_{\infty }\right)})\exp\;(\frac{-K_{g}^{G}}{T_{c}\left(T_{m}^{0}-T_{c}\right)f})$$

where U* is the activation energy for transporting segments to the crystallization front (a universal value is taken as 1500 cal·mol^−1^), *R* is the gas constant with a value of 8.314 J·mol^−1^·*K*^−1^ and *G*_0_ is a constant. *T_c_* is the crystallization temperature. *T_m_*^0^ is the equilibrium melting point and the *T_m_*^0^ value was shown in Table 1 that summarizes the parameters related to the crystallization and melting behavior of the studied PEO PNCs. *f* is the temperature correction factor defined as $f=2T_{c}/\left(T_{m}^{0}+T_{c}\right)$. $T_{\infty }$ is the temperature associated with chain dynamics cease and usually taken as $T_{\infty }=T_{g}-30K$ ($T_{\infty }$ value was shown in Table 1). Fitting the data (converted to the liner formula as *lnG* + U*/(*R*(*T_c_* – *T*_∝_)) vs. 1/$T_{c}\left(T_{m}^{0}-T_{c}\right)f$, as shown in Figure A5 of Appendix E) by the LH theory in Figure 3e allows the prediction of the secondary nucleation energy barrier $K_{g}^{G}$, as shown in Table 1.

Obvious retardation in the growth rate (G) was obtained in Figure 3e with the presence of NPs. Bare SiO_2_ tends to form aggregations at a higher content as 24 wt% (results were confirmed by TEM and SAXS measurement [30]), which may cause the polymer chains to be confined in the restricted space [50]. It is considered that the reduction in the G is mainly linked with the geometric constraints within NPs [51] at a considerably high content. In the case of PEO/PEG-*g*-SiO_2_ with a low *P*/*N* = 0.425, PEO nanocomposites exhibit the lowest G within the measured *T_c_* range. The results may partially relate to an increase of the interfacial interaction under a good dispersion state. Kumar et al. [31] studied the effects of unimodal and bimodal grafted SiO_2_ with various dispersion states on the PEO spherulitic growth and they found the decrease of the growth rate was mainly caused by the increase in the nanocomposite viscosity, which finally hindered the transport of crystallizable segments to the crystalline growth front [34,51,52,53].

**Figure 4:**
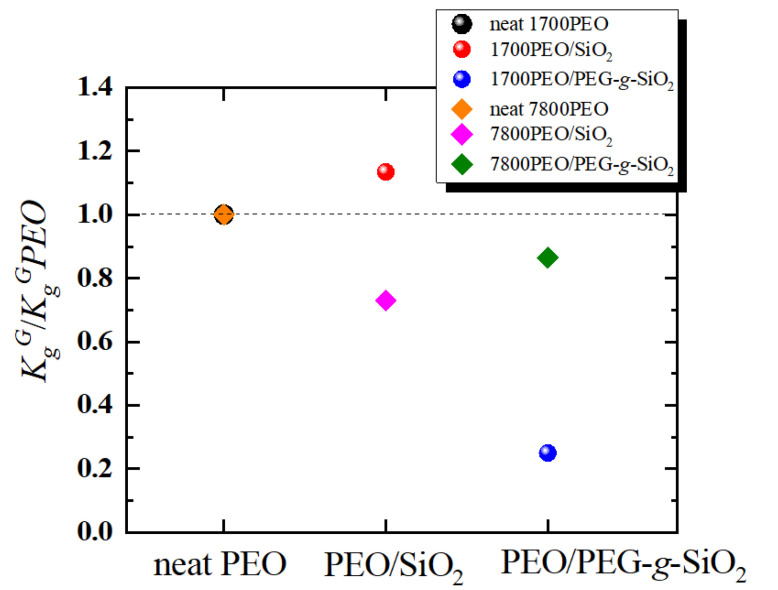
Changing tendency of each PEO nanocomposite in secondary nucleation energy barrier $K_{g}^{G}$ vs. $K_{g}^{G}PEO$.

Figure 4 shows the secondary nucleation energy barrier of the PEO nanocomposites, $K_{g}^{G}$ normalized by the neat PEO, $K_{g}^{G}PEO$. It is clearly implied that the addition of PEG-*g*-SiO_2_ obviously decreases the energy barrier in spherulitic growth. In the case of 1700PEO/SiO_2_, $K_{g}^{G}$ exhibits larger value than that of neat PEO. It means that more energetic requirements are needed for secondary nucleation. Table 2 summarizes the $K_{g}^{G}$ studied in different systems, a decrease of $K_{g}^{G}$ values can be observed with the addition of bare nanoparticles and polymer grafted nanoparticles in PCL nanocomposites [27,54]. The phenomenon that the addition of nanofillers can lower the energetic requirement in the secondary nucleation was also reported in PEO nanocomposites combined with unmodified SiO_2_ NPs (NPs radius = 7 nm) [50], as shown in Table 2. One special case was reported by Kumar et al. [31,55], who shows a relatively minimal change in $K_{g}^{G}$ value with different NPs and loadings, as shown in Table 2. Moreover, they found that the spatial dispersion of the unimodal or bimodal amorphous polymer chains grafted NPs mainly impacts the chain diffusion [31].

### 3.3. Overall Isothermal Crystallization Behavior

The overall isothermal crystallization behavior was probed by the DSC measurements in which both primary nucleation and crystal growth are considered [56,57]. Avrami equation was employed to understand the primary crystallization process. It provides an efficient analytical method to describe the spherulitic nucleation and growth at the early stages of the impingement [27,58,59,60]:

(3) $$1-X_{c}\left(t-t_{0}\right)=\exp\left[-K{\left(t-t_{0}\right)}^{n}\right]$$

where *t* is the crystallization time, *t*_0_ is the induction time, and *n* is the Avrami index. *K* is overall crystallization constant. *X_c_*(*t*) is the relative crystallinity at time *t*.

**Figure 5:**
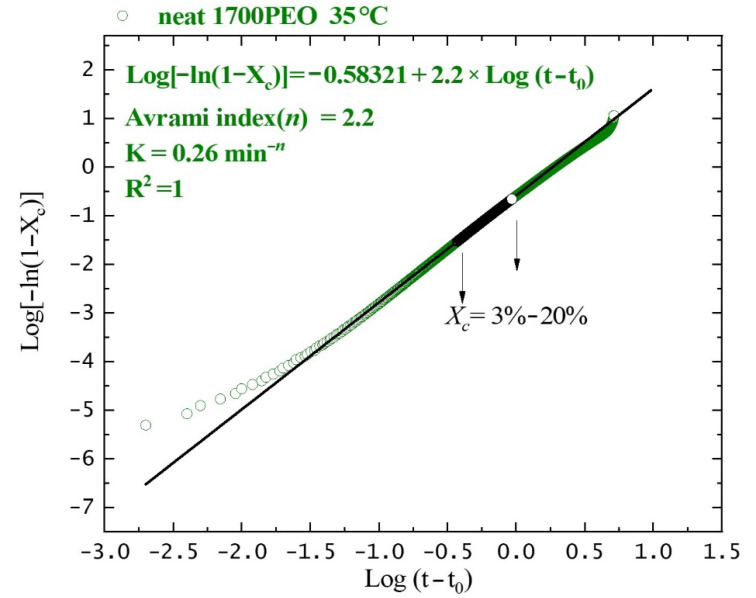
Isothermal crystallization behavior explored by DSC. Plots of relative crystallinity vs. crystallization time for neat 1700PEO at 35 °C. The solid lines are the fittings according to Equation (3).

**Figure 6:**
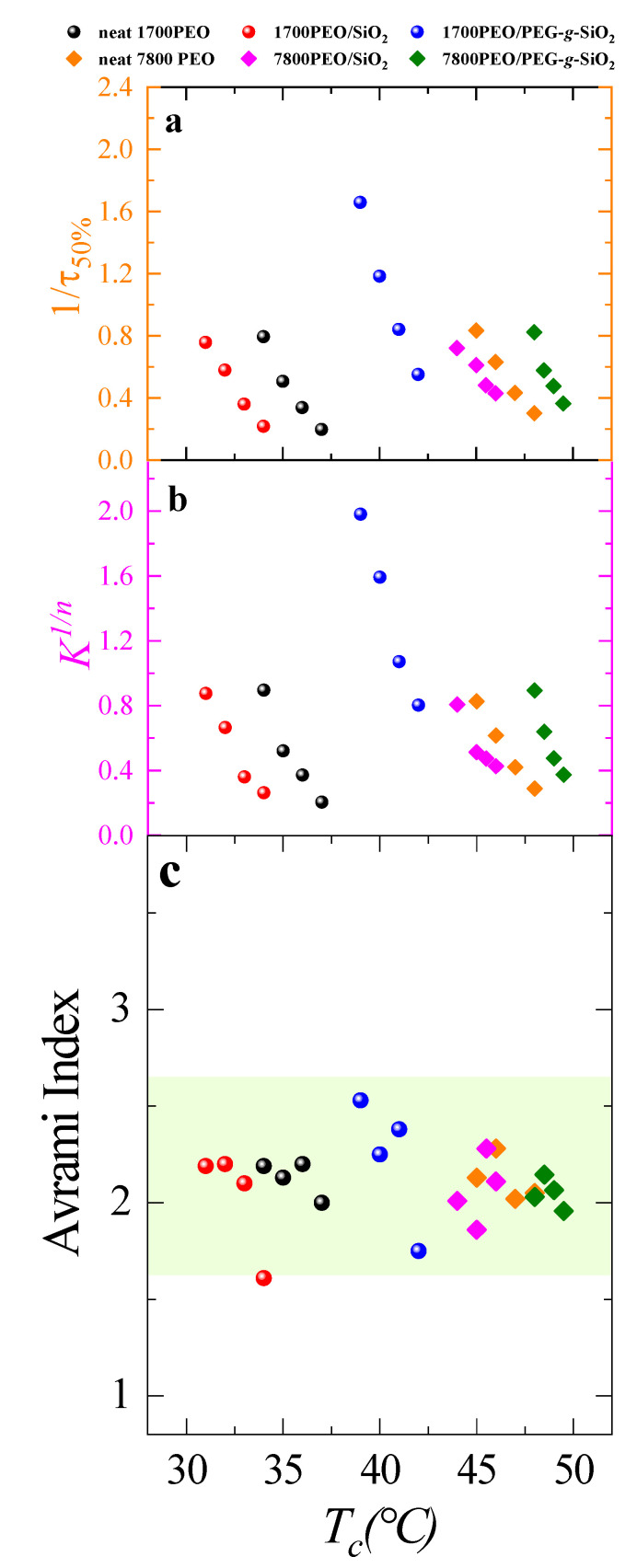
(**a**) The inverse of half crystallization time 1/*τ_50%_* as a function of crystallization temperature, *T_c_*. (**b**) K*^1/n^* as a function of *T_c_*. (**c**) Avrami index *n* as a function of crystallization temperature, *T_c_*.

Figure 5 shows the relative crystallinity of the neat PEO changing with the crystallization time at *T_c_* = 35 °C. The Avrami fittings correspond to the primary crystallization process covering 3%–20%. Figure 6a shows the inverse of half-crystallization, *1*/*τ_50%_* (which represents the overall crystallization rate containing both nucleation rate and growth rate) as a function of the isothermal crystallization temperature, *T_c_*. The overall crystallization rate constant K, obtained from the Avrami equation with a unit of min^−n^, is directly related to the Avrami index, *n*. To make a direct comparison of K in the same units, K was normalized by elevating to the power *1*/*n*. Figure 6b shows the *K*^1/*n*^ (with a unit of min^−1^, which also implies the overall crystallization rate) vs. *T_c_*. It was found that the values of *K*^1/*n*^ predict the trend of experimental data points (*1*/*τ_50%_* vs*. T_c_*) in Figure 6a, which indicates the Avrami theory can adequately fit the data. Figure 6c shows the Avrami indexes, *n* as a function of crystallization temperature. The Avrami index values are very similar for neat PEO and PEO nanocomposites, which are around 2.5 in the tested *T_c_* range, indicating that the PNCs formed spherulites instantaneous [61]. The obtained *n* values reflect the superstructures formed are slightly influenced by the incorporation of SiO_2_.

It is clear that the presence of PEG-*g*-SiO_2_ can obviously accelerate the overall crystallization rate both in the case of *P*/*N* = 0.425(with matrix *M_n_ =* 1700 g/mol) and *P*/*N* = 1.95 (with matrix *M_n_ =* 7800 g/mol). The overall crystallization rate follows the order as: PEO/PEG-g-SiO_2_ > neat PEO > PEO/SiO_2_. Previous studies have shown the high enhancement of the crystallization rate in nanocomposites with the addition of PGNPs [25,34,46], and one possible rationale for these results is attributed to more nucleation sites under the good dispersion of the PGNPs. In our work, as described above, the *T_m_* (as shown in Table 1) of the PEO matrix are almost unaltered by the presence of any SiO_2_ nanoparticle, while the PGNPs endow a high nucleation ability to SiO_2_ nanoparticles, resulting in a marked enhancement of *T_c_*. The surface decoration of the PEG chains remarkably improves the interactions between PGNPs and PEO matrix. Better compatibility of the PEG-*g*-SiO_2_ with PEO matrix compared to the bare SiO_2_ generates notable nucleation sites. Thus, we speculate that the strongly stretched grafted crystallizable chains coupling to a wetting brush interface allows the matrix chains to be templated by the surface chains [39]. In the case of PLOM results, the addition of PEG-g-SiO_2_ notably hindered the spherulitic growth rate. Therefore, the significant increase of the overall crystallization rate should be dominated by the increase of nucleation density.

## 4. Conclusions

We have investigated the isothermal crystallization kinetics of PEO nanocomposites (PEO/SiO_2_ and PEO/PEG-*g*-SiO_2_) using DSC and PLOM techniques. The key conclusion of our study is that the addition of PEG-*g*-SiO_2_ in PEO matrix can alter the primary nucleation and the spherulitic growth rate. Compared to the unmodified SiO_2_, the better dispersion of PEG-*g*-SiO_2_ at lower *P*/*N* shows a stronger nucleation effect and elevates the nucleation density, thus resulting in a marked enhancement in the crystallization rate. On the other hand, the spherulitic growth rate of PEO nanocomposites was significantly retarded.

The results are quite different from the bare silica NPs or NPs grafted with amorphous brushes, in which the addition of NPs does not affect the secondary nucleation energy barrier. Thus, we believe that the nature of the graft chains also plays a crucial role in the crystallization behavior of semi-crystalline polymer nanocomposites.

## Figures and Tables

**Table 1:** Parameters related to the crystallization and melting behavior of the studied PEO nanocomposites.

Sample	*T_c_* (°C)	*T_m_* (°C)	^a^ Δ*H_m_* (J g^−1^)	*X_c_* (%)	*T_m_*^0^ (°C)	*T_g_* (°C)	$T_{\infty }$ (°C)	^b^*K_g_^G^* (K^2^)	^c^ *R* ^2^
neat 1700PEO	28.5	50.4	150.1	73.2	50.8	−45.9	−75.9	5.2 × 10^4^	0.99
1700PEO/SiO_2_	25.9	49.1	159.2	77.6	48.8	−35.7	−65.7	5.9 × 10^4^	0.99
1700PEO/PEG-*g*-SiO_2_	35.1	50.4	182.9	89.2	50.4	−43.6	−73.6	1.3 × 10^4^	0.99
neat 7800PEO	39.2	62.3	171.9	83.8	62.2	−40.3	−70.3	3.7 × 10^4^	0.99
7800PEO/SiO_2_	39.1	60.0	163.9	79.5	61.1	−36.7	−66.7	2.7 × 10^4^	0.99
7800PEO/PEG-*g*-SiO_2_	43.1	61.1	170.4	83.1	62.5	−37.3	−67.3	3.2 × 10^4^	0.96

^a^ The melting enthalpy of PEO nanocomposites was obtained by the non-isothermal crystallization process and normalized by the weight of PEO matrix. ^b^ *K_g_^G^* was obtained by PLOM measurements. ^c^ *R*^2^ represents the correlation coefficient as fitting to Equation (2). The value of Δ*H_m_*^0^ for PEO (melting enthalpy of 100% crystalline PEO) is 205 J/g [44].

**Table 2:** Secondary nucleation energy barrier $K_{g}^{G}$ studied in different nanocomposites.

System 1 [55]	PEO	^a^*V*_PEO_/*SA*_NP_ = 78	*V*_PEO_/*SA*_NP_ = 16	*V*_PEO_/*SA*_NP_ = 139
*K*_g_^G^ (10^4^ K^2^)	13.0	13.1	13.1	12.2
System 2 [50]	PEO	1 wt% SiO_2_/PEO	5 wt% SiO_2_/PEO	9 wt% SiO_2_/PEO
*K*_g_^G^ (10^4^ K^2^)	3.65	3.53	3.49	3.36
System 3 [54]	PCL	^b^ PCL–CNW	^c^ PCL–MFC	
*K*_g_^G^ (10^4^ K^2^)	14	6.3	5.5	
System 4 [27]	^d^ L-PCL	^e^ L-PCL/SWNT-ODA	^f^ L-PCL/MWNT-g-PCL	
*K*_g_^G^ (10^4^ K^2^)	11.2	10.4	8.7	

^a^ V_PEO_/SA_NP_ represents the ratio of PEO volume (V_PEO_) to NPs surface area (SA_NP_). ^b^ PCL–CNW represents PCL with surface modified sisal nanowhiskers (CNW) and ^c^ PCL–MFC represents PCL with microfibrillated cellulose (MFC). ^d^ L-PCL represents linear poly(ε-caprolactones). ^e^ L-PCL/SWNT-ODA represents PCL with octadecylamine functionalized single wall CNTs. ^f^ L-PCL/MWNT-g-PCL represents PCL with linear PCL grafted multiwall carbon nanotubes.

## Data Availability

Data is contained within the article.

## Appendix C. Equilibrium Melting Temperature of the PEO Nanocomposites

*T_m_* is the melting point of the specimens after complete crystallization at different isothermal crystallization temperatures. The equilibrium melting temperature *T_m_* °C, corresponding to a hypothetical infinitely thick crystal, can be obtained by the direct or indirect extrapolation of *T_m_* data to *T_m_* = *T_c_*.
